# Proteomic Biomarkers for the Detection of Endometrial Cancer

**DOI:** 10.3390/cancers11101572

**Published:** 2019-10-16

**Authors:** Kelechi Njoku, Davide Chiasserini, Anthony D. Whetton, Emma J. Crosbie

**Affiliations:** 1 Division of Cancer Sciences, School of Medical Sciences, Faculty of Biology, Medicine and Health, University of Manchester, 5th Floor Research, St Mary’s Hospital, Oxford Road, Manchester M13 9WL, UK; kelechi.njoku@manchester.ac.uk; 2 Department of Obstetrics and Gynaecology, Manchester Academic Health Science Centre, Manchester University NHS Foundation Trust, Manchester M13 9WL, UK; 3 Stoller Biomarker Discovery Centre, Institute of Cancer Sciences, Faculty of Medical and Human Sciences, University of Manchester, Manchester M13 9PL, UK; davide.chiasserini@manchester.ac.uk (D.C.); tony.whetton@manchester.ac.uk (A.D.W.)

**Keywords:** endometrial cancer, diagnostic biomarkers, proteomics, mass spectrometry

## Abstract

Endometrial cancer is the leading gynaecological malignancy in the western world and its incidence is rising in tandem with the global epidemic of obesity. Early diagnosis is key to improving survival, which at 5 years is less than 20% in advanced disease and over 90% in early-stage disease. As yet, there are no validated biological markers for its early detection. Advances in high-throughput technologies and machine learning techniques now offer unique and promising perspectives for biomarker discovery, especially through the integration of genomic, transcriptomic, proteomic, metabolomic and imaging data. Because the proteome closely mirrors the dynamic state of cells, tissues and organisms, proteomics has great potential to deliver clinically relevant biomarkers for cancer diagnosis. In this review, we present the current progress in endometrial cancer diagnostic biomarker discovery using proteomics. We describe the various mass spectrometry-based approaches and highlight the challenges inherent in biomarker discovery studies. We suggest novel strategies for endometrial cancer detection exploiting biologically important protein biomarkers and set the scene for future directions in endometrial cancer biomarker research.

## 1. Introduction

Endometrial cancer (EC) is the most common gynaecological malignancy in the western world and the sixth most common cancer in women worldwide. Over 300,000 new cases are diagnosed annually, accounting for about 8.2% of the worldwide incidence of cancer in women. Its incidence varies across regions and is rising as life expectancy increases [1,2]. In the United Kingdom, it is the fourth most common women’s cancer with more than 9000 incident cases every year [3]. When diagnosed at an early stage, EC is highly curable and has excellent overall 5-year survival rates [4]. Delayed diagnosis contributes to advanced stage at presentation and poor survival. In Europe, an estimated 12,000 women die of EC annually of whom >2000 live in the United Kingdom [2,5].

Endometrial cancers have traditionally been classified into two histological categories: type 1 and type 2 (Bokhmans dualistic model) [6]. Type I tumours make up 80–90% of endometrial cancers and are oestrogen responsive, have a favourable prognosis, and may be preceded by a precancerous condition (atypical hyperplasia). Type II tumours, on the other hand, account for only 10–20% of endometrial cancers and are usually oestrogen independent, high grade and clinically aggressive [6,7]. A recent and pragmatic classification of EC based on a multiplatform (genomic and transcriptomic) analysis categorizes EC into four distinct molecular subtypes of prognostic relevance: polymerase episilon (*POLE)* ultramutated, microsatellite unstable (MSI), copy number low and copy number high, and has been validated in multiple studies [8,9].

Postmenopausal bleeding (PMB) is the most frequent symptom of EC and is experienced as blood in urine by some women. Only 5–10% of women with PMB have EC, however. Postmenopausal women with vaginal bleeding undergo various tests to exclude EC, including transvaginal ultrasound scan (TVS), outpatient hysteroscopy (OPH) and endometrial biopsy (EB) [10]. While these procedures are expensive and sometimes difficult to perform, their diagnostic utility for EC is mainly limited by poor specificity (TVS) and unacceptable levels of invasiveness and discomfort (OPH, EB) [4,10]. The measurement of endometrial thickness (ET) with TVS for instance, although minimally invasive and highly sensitive for EC detection in postmenopausal women, is plagued by a markedly low specificity as multiple benign pathologies, specifically polyps, intracavitary fibroids or blood clot artefacts create the appearance of a thickened endometrium [4,10]. Thus all women with a thickened endometrium undergo further invasive investigations to establish a diagnosis [11]. Endometrial biopsy, the gold standard for the diagnostic evaluation of women with suspected EC, can sometimes miss focal pathologies, especially when done blindly using office-based sampling devices such as pipelle, and is not only particularly painful in nulliparous women but also has a high risk of insertion failure [4,12]. Hysteroscopy with directed biopsy, on the other hand, has better diagnostic sensitivity but is expensive, has a high failure rate in the outpatient clinic and over 30% of women experience severe pain or a vasovagal episode during its completion. There is the theoretical risk of disseminating cancer cells into the peritoneum and rarely life threatening complications ensue, for example uterine perforation [4,13]. The ideal EC detection tool should be simple, non-invasive and have the ability to reliably detect all ECs at the earliest stage with few false positives or negatives. Such a tool may also be used to screen high risk asymptomatic women, for instance women with Lynch syndrome, who have a high lifetime risk of developing EC. Early detection could enable conservative management options to be offered to young women, especially those yet to complete childbearing and morbidly obese women in whom surgery is potentially hazardous [10]. Diagnostic biomarkers that identify specific subtypes of EC, for example *POLE* ultramutated EC, would also provide prognostic and predictive information, and could be used to monitor response to therapy and detect recurrent disease.

## 2. Search for Endometrial Cancer (EC) Diagnostic Biomarkers Using High-Throughput Technologies

In recent years, high-throughput technologies have demonstrated potential as large-scale biomarker discovery platforms. These include genomic, transcriptomic, proteomic, metabolomic and imaging analyses [14]. The use of genomics dates back to 2001 when a sequence of the human genome was decoded. Genomics provides information about the full set of genes within a cell, rather than focusing on individual genes, and has enormous potential to enable the discovery of novel biomarkers and diagnostic tests [15,16]. In the integrated genomics characterisation of EC by the Cancer Genome Atlas Network, multiple EC-defining molecular defects were identified [17]. Based on these data, several studies using next generation sequencing and array based technology have searched for somatic mutations in various biological specimens with the view to developing novel EC diagnostic tests. Nair and colleagues, for example, using ultra-deep next-generation sequencing (NGS) identified somatic mutations in DNA extracted from both cell pellets and cfDNA fraction of uterine lavage samples in women with EC [18]. Lim et al. used NGS on cervical swab samples from EC patients to identify specific genes with potential to serve as markers for EC detection [19]. Both studies were, however, limited by high false positive rates.

Despite initial optimism that genomics could revolutionise clinical diagnostics, it became clear that knowledge of our genome alone is insufficient to elucidate all relevant disease-specific interactions at the molecular level, perhaps not surprising as environment plays such a major role in disease causation [16]. Genomic analysis is limited by its inability to provide complete information on cellular, subcellular, and intercellular functions [20]. These limitations spearheaded rapid progress in interdisciplinary systems biology that integrate genomic/epi-genomic, transcriptomic, proteomic and metabolomic data. In comparison to the genome, the epigenome is much more dynamic and reflects multiple functional states separated in time and space. Epigenetic modifications are reversible and heritable changes in gene function occurring in the absence of changes in the nucleotide sequence [21]. Epigenomic dynamics are governed in part by reversible covalent modifications such as DNA methylation and histone tail modification [22,23]. The silencing of tumour suppressor genes by site specific DNA hypermethylation is the main mechanism of epigenome-induced carcinogenesis. Global hypomethylation, a frequently observed phenomenon in human cancers, can result in chromosomal instability and oncogene activation [24,25]. Although epigenetic studies in EC are still at an early stage of development, our understanding of the methylation changes underlying the EC phenotype continues to improve as genome-wide profiling techniques of human DNA methylation continue to develop [23]. Jones et al., in an epigenome-wide methylation analysis of >27,000 CpG sites in 64 EC tissues and 23 controls, identified *HAND2* DNA methylation as a possible biomarker for EC but this is yet to be validated [25]. Wentzensen and colleagues, using DNA methylation profiling, identified 114 CpG sites showing methylation differences between EC cases and controls of which eight were selected for validation [26]. This study was limited by imbalances in the age distribution of the two groups. Similarly, Huang and colleagues identified three hypermethylated genes, *BHLHE22, CDO1*, and *CELF4*, in the cervical scrapings of women with EC with sensitivity and specificity of 83–96% and 78–96% respectively [27]. The search for biomarkers using an epigenetics-based approach has important limitations, however. DNAme assays are complex and require significant care in primer design and optimization. Widely used assays like methylation-specific polymerase chain reaction (PCR) are either non-quantitative or produce semi-quantitative data with poorly defined cut-offs based on complex ratios of test and control genes. Such test assays may be useful in the research setting but have poor reproducibility in routine clinical settings [22].

High throughput technologies have also been applied in the large scale study of RNA in what is known as transcriptomics. The study of the transcriptome, the complete set of RNA transcripts produced by the genome in a specific cell, allows for the characterisation of genetic expression at the mRNA level [28]. In contrast to DNA, actively transcribed RNA is highly dynamic and reflects the diversity of cell types and their regulatory mechanisms [29]. Cancer cells have aberrant transcriptional patterns that play a role in their growth advantage. RNA sequencing can identify and quantify differentially expressed genes and thus has great potential in the search for disease-relevant biomarkers. Interrogating the EC transcriptome in the cancer genome atlas (TCGA) did not, however, provide enough transcriptome-wide information on differentially expressed genes in EC versus normal endometrial tissue [17]. Shi and colleagues using RNA-seq reported IGSF9 to be over-expressed in EC compared to normal healthy controls [30]. This was based on a small retrospective cohort of patients and is yet to be validated. Similarly, Jiang et al. using Solexa sequencing identified miR-887-5p as a potential biomarker of EC, a finding yet to be validated [31]. Although transcripts are dynamic, they do not truly reflect the functional phenotype of a cell as they are not their final genetic products. There is also limited correlation between mRNA levels and encoded proteins. Because the proteome better reflects the dynamic state of cells, tissues and organisms, proteomics has great potential to yield actionable and clinically relevant biomarkers [32,33].

## 3. Proteomic Approaches for EC Detection

Proteomic technologies in combination with computational analyses have emerged as powerful tools for biomarker discovery based on the simultaneous analysis of thousands of proteins. They are able to identify molecular fingerprints based on protein pathways or diagnostic algorithms that rely in part on protein quantification [20]. Proteomics characterises not only all the proteins within a cell, but also their various isoforms and modifications, including the interactions between them. The identification of proteins with altered expression in cancer is possible because of advances in sample preparation, protein separation and mass spectrometry (MS)-based analysis [20].

Two-dimensional gel electrophoresis (2-DE), protein arrays and MS, in conjunction with advanced bio-informatics are all valuable tools for protein biomarker discovery. 2-DE technology allows for a global profiling of a sample proteome by the simultaneous resolution of hundreds to thousands of proteins on a single gel. However, it is limited by its inability to resolve several classes of proteins (i.e., transmembrane proteins) and by its low throughput that does not allow application to clinical settings [34]. Protein arrays, on the other hand, make use of specific antibodies of known affinity and allow for the observation of the expression of hundreds to thousands of proteins. Prior knowledge of the targets is needed in order to utilise this technique for biomarker detection. Protein arrays are limited by the need for large-scale production of good quality antibodies using recombinant platforms which is challenging due to the complexity of protein expression and purification processes [35,36].

Protein biomarkers have a number of advantages over RNA, DNA and metabolites for EC detection. First, a large part of the human proteome is detectable in easily accessible biological fluids, thus enhancing their potential clinical utility. Second, they are easily isolated and quantified using commonly used laboratory tests such as enzyme-linked immunosorbent assay (ELISA) and immunohistochemistry, again, enhancing their translational potential [37].

### Mass Spectrometry-Based Proteomic Approaches for EC Detection

Mass spectrometry (MS) has become the technique of choice for identifying and quantifying proteins in biological samples and has revolutionized the study of proteins, enabling comprehensive analysis of complex human samples in order to better understand normal physiology and pathogenic mechanisms of disease [20]. The main principle behind MS-based analysis is the ionization of chemical compounds into charged molecules or molecule fragments and the subsequent measurement of their mass-to-charge ratios with confirmation of identity by fragmentation-based sequencing [16].

The two main MS-based proteomic approaches currently used in biomarker discovery research are: top-down and bottom-up approaches. While the bottom-up approach is more frequently used and involves the proteolytic digestion of separated proteins in biological specimens and subsequent MS-based identification of the peptide fragments followed by protein inference, the top down approach derives protein sequence information directly from the analysis of intact proteins without the need for enzymatic digestion [16,38,39]. The top-down approach, however, is limited by the complexity of the MS/MS spectra of large proteins that are often difficult to interpret and require the development of new algorithms for signal deconvolution [16]. Proteomic studies take advantage of the “shotgun” technology, which is a bottom-up strategy that involves an initial proteolytic digestion of the entire sample, subsequent separation of the protein peptide mixture by liquid chromatography (LC) and final identification using tandem mass spectrometry (MS/MS) [39]. Shotgun proteomic approaches allow for the detection of over 10,000 proteins in a single run [32]. The quantification of proteins from such experiments is done by the use of stable isotopes or by label-free methods. Isotope labelling has a better precision and accuracy in comparison to label-free techniques. However, label-free techniques have become more popular as they are a lot cheaper, less difficult to undertake and less time consuming [38]. As such, with label-free techniques, large numbers of samples can be analysed in clinical settings. Traditional liquid chromatography–mass spectrometry (LC–MS) (shotgun) techniques are based on a data-dependent acquisition (DDA) strategy in which a number of peptide signals are selected for fragmentation based on their relative abundance and are subsequently matched to a pre-defined database [40]. This stochastic approach can result in loss of valuable information on low-abundant peptides and lead to incomplete datasets, with high number of missing values [41]. In contrast, data-independent acquisition (DIA) fragments every single peptide in a sample within defined mass windows, thus allowing for a more sensitive and accurate peptide profiling, reducing the number of missing values and increasing reproducibility [38,39,40]. Targeted approaches focus on the analysis of a pre-selected group of peptides/proteins and have several advantages in terms of reproducibility and precision [16,39]. An emerging technology of high precision and accuracy that enables label-free quantification of proteins is the sequential window acquisition of all theoretical mass-spectra (SWATH–MS). SWATH–MS uses data-independent acquisition methods to provide a highly comprehensive and reproducible analysis of proteins and peptides in complex biological samples [42]. SWATH–MS is able to create a digital proteomic map that can be stored and used for re-analysis. Such permanent digital inventory has several advantages in terms of being cost and time-efficient as samples do not need to be re-prepared and re-run multiple times where re-analysis is needed. In addition, they have value in situations where physical sample storage space is an issue or where samples are limited in quantity or are at a risk of degradation [42]. Another emerging technology with promise, especially for tissue-based biomarker discovery, is matrix-assisted laser desorption/ionization (MALDI) imaging. This label free in situ technique allows for a direct profiling of proteins and their abundance in thin tissue sections and enables the visualisation of proteins and peptides in a spatial context and correlation of molecular information with traditional histology [43,44].

## 4. Proteomics Biomarkers for EC Detection

Biological specimens that have previously been investigated in the search for EC diagnostic biomarkers include serum/plasma, hysterectomy specimens, uterine aspirates/tissue biopsies, uterine lavage samples and urine (Table 1) [45]. While systemic fluids such as blood are undoubtedly the preferred source of biomarkers as they are easily accessible using minimally invasive procedures, their use for diagnostic biomarker discovery is limited by the low quantity of tumour-related signal in the circulation in the early phases of a disease [45,46]. As such, there are issues around sensitivity and specificity with the use of such fluids for early cancer detection (Table 2). Hysterectomy, endometrial biopsy specimens and uterine lavage samples, on the other hand, are viable sources of cancer-derived proteins and are a less challenging matrix for proteomic analysis in comparison to blood due to the much lower protein dynamic range [47]. They are however limited by the invasiveness of sample acquisition (Table 1) [45]. Importantly, the presence of a biomarker in tissue does not necessitate its expression in less-invasive samples like blood and urine. However, given the anatomical continuity of the uterine cavity with the lower genital tract, it is plausible that such biomarkers may be expressed in proximal fluids sampled using less-invasive strategies (Table 1). Studies exploring this possibility are urgently needed as they are likely to yield clinically relevant biomarkers.

Effective sample preparation is critical for the success of EC biomarker discovery [48]. The initial sample preparatory step in proteomic studies is the extraction of proteins through lysis of biological materials and is essential for the isolation of proteins from endometrial tissue or cellular materials. Laser capture micro-dissection (LCM) is often employed in tissue-based proteomic studies and enables the microscopy-guided isolation of specific tissue regions or cell types, thus preserving relevant spatial information [49]. While there is no standard way to extract proteins from samples, the choice of a lysis strategy is often based on the protein target as well as the sample size, location and required yield of the protein of interest, proposed downstream MS applications and experience of the researcher [48,50]. For biological fluids like plasma, the sample preparation technique is slightly different and often involves depletion of high abundant proteins, solubilisation and concentration of the samples [32].

### 4.1. Proteomic Analysis of Blood for EC Detection

**Figure 1:**
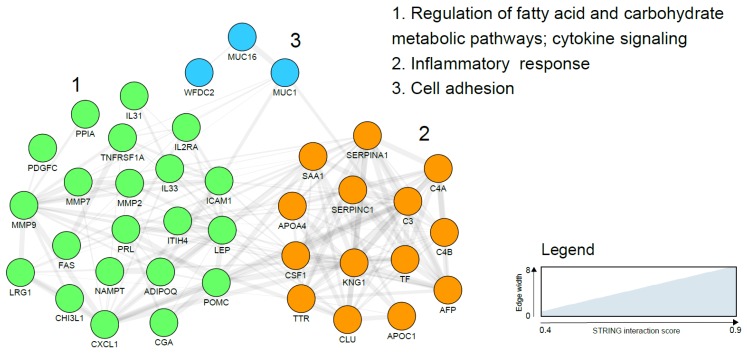
Endometrial cancer blood based biomarker correlation network based on the search tool for the retrieval of interacting genes/proteins (STRING) network analysis using gene names and visualised with the Cytoscape software (https://cytoscape.org/). Line thickness indicates strength of the interactions. Protein biomarkers were clustered using the markov cluster (MCL) algorithm and subjected to functional enrichment. On the right, the biological processes describing the functions of the candidates are indicated. No significant interactions were reported for Dickkopf-related protein 3 precursor (DKK3), Sperm associated antigen-9 (SPAG 9), Alpha-1-beta glycoprotein (AIBG) and Growth differentiation factor 15 (GDF-15) and, therefore, are not included in the final network.

Peripheral blood is the prototypical liquid biopsy with materials such as circulating tumour cells (CTCs), circulating tumour DNA (ctDNA), proteins and extracellular vesicles all having the potential to serve as biomarkers. Several blood-based protein biomarker candidates for endometrial cancer detection have been reported using a wide variety of molecular approaches and are broadly categorised as: hormones (prolactin (PRL), thyroid stimulating hormone (TSH), adrenocorticotrophic hormone (ACTH), follicle stimulating hormone (FSH), cancer associated antigens (CA125, CA15-3, CA72.4), adipokines (leptin and adiponectin), complement factors (C3, C4A, C4B), plasma glycoproteins (alpha-1-beta glycoprotein (AIBG), antithrombin III **(**SERPINC I), chitinase-3 like protein1 (YKL-40)), plasma lipoproteins (serum amyloid A (SAA)), apolipoproteins (ApoA), enzymes (matrix metalloproteinases such as MMP-2, MMP-7, MMP-9), enzyme inhibitors (human epididymis protein 4 (HE4), alpha-1 antitrypsin **(**SERPINA1), and growth factors (growth differentiation factor 15(GDF-15), vascular endothelial growth factor (VEGF)), among others (Table 2). There is, at present, insufficient evidence to support the use of any of these biomarkers, either in isolation or in combination for EC diagnosis. Translation into routine clinical use has been impossible due to the inconsistency of study findings, sub-optimal accuracy and lack of robust validation of most biomarker candidates. Some markers are surrogates for EC risk factors rather than being diagnostic of EC per se and are unlikely to demonstrate sufficient accuracy when used in a population at modest or low risk of EC. As an example, the prevalence of obesity, the strongest modifiable risk factor for EC, is likely to systematically differ between cohorts of women with and without EC and circulating adipokines such as leptin, adiponectin and visfatin may only reflect this difference [51,52]. Obesity related metabolic and endocrine conditions may also explain the altered levels of other putative markers such as prolactin [53] and TSH [54]. The mechanisms underpinning the association between many other reported biomarker candidates such as AFP and EC are unclear and further studies are needed. Some of the most reported serum EC biomarkers such as AIBG, SERPINA1 and Apo A1 are high abundance and inflammation-related plasma proteins, limited by their lack of specificity for EC. The two most studied EC biomarker candidates are HE4 (WFDC2) and CA125 (MUC 16), both of which have also been reported in EC tissue specimens and validated in independent cohorts [37,55]. Li and colleagues, in a meta-analysis of 23 studies involving more than 4000 participants, reported a pooled sensitivity and specificity of 0.65 (95% confidence interval (CI) 0.56–0.73) and 0.9(95% CI (0.8–0.95) respectively for HE4 [55]. These findings should be interpreted with caution given the substantial heterogeneity across the studies. Importantly, the sub-optimal sensitivity has important clinical implications including risk of false reassurance and knock on consequences for delayed presentation, advanced stage at diagnosis and poor survival. CA125 (MUC 16), a tumour marker commonly used in the management of ovarian cancer, has also failed to demonstrate sufficient accuracy for EC detection (sensitivity of 17.8–52.6% and specificity of 33.35% to 95%), even when combined with HE4 (sensitivity of 57–76% and specificity 90–100%) [37]. In an attempt to further improve the diagnostic accuracy of combined HE4 and CA125, Knific and colleagues incorporated clinical data, specifically BMI, in their algorithm and reported a sensitivity of 66.7% and specificity of 84.6% [56]. Future studies on HE4 and CA125 should aim to incorporate additional protein markers with the view to producing a robust panel with sufficient diagnostic accuracy. Of the several serum-based biomarker candidates identified by Yurkovetsky and colleagues, prolactin demonstrated the strongest discriminatory ability for EC detection with an overall sensitivity of 98.3% and specificity of 98% [57]. The upregulation of prolactin in EC has been postulated to be due to an increased secretion by stromal cells in response to tumour growth and differentiation. In addition, prolactin modulates angiogenesis, an essential component of tumorigenesis [58]. However, increased prolactin has also been observed in ovarian, pancreatic and lung cancers, thus limiting its utility as a specific biomarker for EC [57,58]. Prolactin, like most hormones, is also known to exhibit a circadian rhythm, further complicating its analysis and study comparisons. In combination with other biomarker candidates, specificity for EC is improved. The panel consisting of prolactin, GH, TSH, eotaxin and E-selectin has shown better accuracy for EC discrimination from ovarian and breast cancers in comparison to prolactin alone [57,59]. Further studies are needed to clarify the role of prolactin in EC diagnosis. Other blood-based biomarker candidates and their reported diagnostic accuracies are summarized in Table 2. A network visualization of the interactions using the STRING database (Search Tool for the Retrieval of Interacting Genes/Proteins) between identified blood biomarker candidates is presented in Figure 1 and clearly shows three functional and biological clusters: regulation of metabolic and cytokine mediated pathways, inflammatory response and cell adhesion (Figure 1).

### 4.2. Proteomic Analysis of Tissue Samples for EC Detection

A variety of proteins have been reported as possible EC diagnostic markers using endometrial tissue and uterine lavage specimens and as described in Table 3. The candidate biomarkers are broadly categorized as: chaperones/heat-shock proteins (hsp10, hsp27, hsp70, hsp71), enzymes (pyruvate kinase (PK), phosphoglycerate kinase (PGK-1), phosphoglycerate mutase 2 **(**PGAM2), alpha enolase (ENO-1)), enzyme inhibitors (alpha-1-antitypsin precursor (SERPINA 1)), calcium-binding proteins (calgranulin(S-100A8/9), calgizzarin (S-100A11), calcyphosine (CAPS)), fatty acid binding proteins (epidermal fatty acid protein (FABP5)) and cytoskeletal proteins amongst others. Studies have been consistent in revealing an overexpression of heat-shock proteins in endometrial cancer tissue specimens [89,91,92,93,94,95]. DeSouza and colleagues, using tandem MS reported an upregulation of chaperonin 10 in EC tissues [89]. This was subsequently replicated in a verification study where the panel of chaperonin 10, pyruvate kinase and alpha1-antitrypsin demonstrated a sensitivity, specificity and positive predictive value of 0.95 each [96]. Heat-shock proteins, also known as molecular chaperones, regulate protein folding, cell signalling and maintenance of the conformation of transduction complexes. They are implicated in tumour cell proliferation and differentiation and are overexpressed in a wide variety of human cancers [91]. The mechanism by which they induce carcinogenesis, however, is not fully understood. It is postulated that physio-pathological features of the tumour microenvironment including changes in oxygen concentration, pH and glucose levels propagate Hsp induction [91]. Regardless of the mechanism underpinning their oncogenic tendency, their potential as EC diagnostic biomarkers is limited by their non-specificity (Table 2) [95,97,98]. In combination with other proteins, however, they are likely to be strong candidates for EC detection and warrant further exploration.

Using targeted MS-based techniques on uterine aspirates from 20 EC cases and 18 non-EC controls, 10 proteins were reported by Martinez-Garcia and collaborators to be differentially expressed and include: myeloperoxidase, E cadherin, alpha enolase, metalloproteinase 9 (MMP9), pyruvate kinase, peroxiredoxin 1, osteopontin, lactate dehydrogenase A, Kunitz-type protease inhibitor and caspase-3, all of which had an AUC greater than 0.9. Four of these proteins; myeloperoxidase, E-cadherin, Kunitz-type protease inhibitor and osteopontin had sensitivity above 80% at 95% specificity [99]. However, it was unclear whether the study was sufficiently powered for biomarker detection. In a further study by Martinez et al., the combination of MMP9 and KYPM in the fluid fraction of uterine aspirates demonstrated a sensitivity of 94% and a specificity of 87% for the detection of EC while the combination of beta-catenin (CTNB1), exportin 2 (XPO2) and macrophage-capping protein (CAPG) demonstrated a 95% sensitivity and 96% specificity for discriminating EC subtypes [100]. Ura et al., using proteomics-based approaches identified four proteins: costars family protein ABRACL, phosphoglycerate mutase 2, fibrinogen beta chain and annexin 3 in the uterine aspirates of endometrial cancers and not in healthy aspirates. This should be interpreted with caution given the small study size. Further verification by Western blot demonstrated the differential expression of only two proteins; costars family ABRACL and phosphoglycerate mutase 2 (PGAM2) [101]. Further studies are needed to validate both ABRACL and PGAM2 as EC diagnostic biomarkers prior to their clinical utility.

In the 2-DE analysis of more than 90 initially identified proteins by Li and colleagues, CYPA demonstrated a 27.2-fold upregulation in EC while FABP5 and CAPS were upregulated 6.5 fold and 3.7 fold, respectively [80]. Although CYPA was initially thought to be predominantly intracellular, studies have shown CYPA to be secreted from cells in response to inflammatory stimulation and to have roles in protein folding, immune response and HIV-1 infection. E-FABP, a member of the fatty-acid binding proteins, is involved in cellular signalling and influences gene expression and cell differentiation. CAPS, on the other hand, has been implicated in cell proliferation and differentiation. These biomarker candidates are yet to be validated and studies investigating their link with EC are needed.

**Figure 2:**
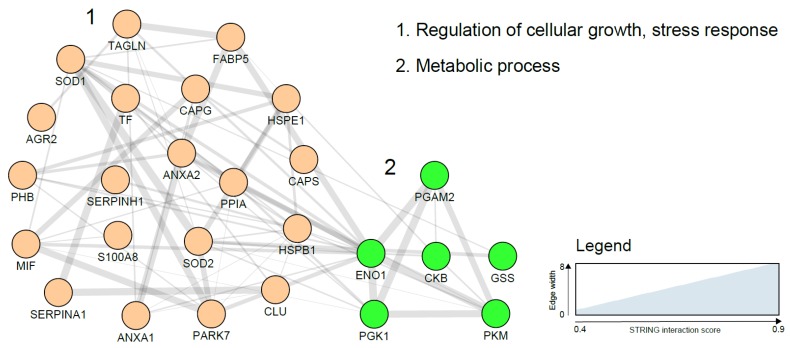
Endometrial cancer tissue based biomarker correlation network based on a STRING network analysis using gene names and visualised with the Cytoscape software (https://cytoscape.org/). Line thickness indicates strength of the interactions. Protein biomarkers were clustered using the MCL algorithm and subjected to functional enrichment. On the right, the biological processes describing the functions of the candidates are indicated. Costars family protein (ABRACL), Desmin (DES), Fibrinogen beta chain (FBG) and polymeric immunoglobulin receptor precursor (PIGR) did not show any previously reported interaction and are not included in the figure above.

A number of glycolytic enzymes have been suggested as potential EC diagnostic biomarkers and include PK and PGKI. Overexpression of these proteins in malignant cells can be explained on the basis of the critical role they play in ATP generation in the glycolytic pathway. In hypoxic states, as is typical of most cancers, the glycolytic pathway allows the cancer cells to meet the higher energy requirements needed for proliferation [102]. A study using MS analysis of EC cells harvested using laser microdissection, identified annexins and peroxiredoxins as over-expressed in EC. Calgizzarin (S100A11), transgelin and several other proteins have been reported to be differentially expressed between EC cases and controls (Table 2). Further studies are needed to not only validate these findings but also elucidate their role in EC carcinogenesis. A network visualization of the interactions among the tissue biomarker candidates using the STRING database is presented in Figure 2 and clearly shows two functional and biological clusters: regulation of cellular growth/stress response and metabolic processes. While metabolic regulation was the predominant cluster in blood-based biomarkers, regulation of cellular growth and stress response was more represented in tissue-based markers. Additionally, tissue-based biomarkers were mainly cellular proteins while secreted proteins abundant in biological fluids dominated the plasma biomarker network.

### 4.3. Proteomic Analysis of Urine for EC Detection

Few studies have investigated urine as a potential source of EC diagnostic biomarkers using proteomic approaches. In the study by Mu et al., urinary levels of Zinc alpha-2 glycoprotein, alpha-1 acid glycoprotein and CD59 were reported to be upregulated in EC cases compared to healthy controls while nebulin was downregulated in EC [109]. Urine is indeed an attractive sample for biomarker discovery as it is cheap, easily accessible using non-invasive methods and can be collected in large amounts and repeatedly at home and in privacy [110] (Table 1). It is also a useful biofluid for proteomic analysis as proteins and peptides excreted in urine are generally stable and less complex in comparison to plasma/serum. However, there is wide variability in urinary protein concentrations as age, diet, genetics and many environmental factors influence the urinary protein profile of each individual [111]. The discovery of urine-based EC biomarkers is dependent on the renal excretion of systemic biomarkers or urinary contamination by uterine biomarkers. Renally excreted EC biomarkers may be limited by the difficulty in finding systemic biomarkers in early disease while those resulting from the contamination of urinary flow by uterine shed biomarkers can be unreliable and inconsistent especially in asymptomatic women. More studies exploring urine-based biomarkers in symptomatic women are needed.

### 4.4. The Ideal EC Proteomic Biomarker

Developing an ideal diagnostic test for EC will require harnessing the potential of a sensitive and reproducible technology with non-invasive sampling methodology. In recent times, there has been growing interest in the use of minimally invasive sampling strategies for EC detection (Table 1). The Pap smear for instance, established as a screening tool for cervical cancer, has so-far failed to show satisfactory performance for EC detection based on cytology [112], although a few proof of concept studies have shown its feasibility for EC diagnosis when combined with genomic or epigenomic biomarkers [113]. With a Tao brush, sensitivity is improved but at the expense of increased invasiveness, reduced acceptability, higher costs and relatively common insertion failure [114]. The Pap smear approach is not without side effects including discomfort of speculum examination but is more acceptable to women than pipelle biopsy or uterine lavage. Cervical scraping and swabs have also been explored in a few studies looking at the levels of CA125 [115] and more studies are needed. Other sampling methods such as vaginal tampons need further exploration, although pilot work suggests they are unappealing or unacceptable to some postmenopausal women, particularly those who are elderly, and less robust for EC detection in women without abnormal vaginal bleeding [116]. Studies investigating the effectiveness of novel approaches that combine non-invasive sampling methodology with high-throughput proteomics for EC detection are urgently needed.

### 4.5. Challenges in Endometrial Cancer Diagnostic Biomarker Validation and Usage

Not all biomarkers identified in the discovery phase reach clinical validation. Erroneous conclusions about the discriminatory ability of a putative biomarker may be due to chance, lack of assay generalizability or bias [117]. Bias is, perhaps, the most important threat to the validation of biomarker studies and can occur at different stages of discovery research depending on the study design and technology used. An important factor that can introduce bias is subject selection [118]. A large number of EC biomarker studies mainly included pre-menopausal women while an even larger number used healthy asymptomatic women as controls. As EC is predominantly a postmenopausal disease, this should ideally be reflected in the control study population. Importantly, controls should include women from a similar at risk population, such as those with PMB. Systematic differences between cases and controls should be avoided as they reduce the likelihood that the discriminatory ability of putative biomarkers are disease-related. Clearly defined eligibility criteria for patient selection are required in order to guide generalisability of study findings.

Another source of bias in biomarker research is pre-analytical variables that have the potential to introduce spurious signals into specimens [119]. Quite frequently, case specimens are collected over time and stored until analysis while control specimens are collected at different time points or at different sites. Storage time variability is known to be a possible source of bias in proteomics-based diagnostic studies [120]. It is important that standard operating procedures are applied to samples with regards to collection, processing, storage and number of freeze/thaw cycles. Samples should ideally be analysed in a blinded manner. If samples cannot be analysed in the same batch, case and control specimens should be mixed during analysis using block randomization and not run in separate batches [119]. 

A sample size that is smaller than the ideal for biomarker discovery increases the possibility of making erroneous conclusions. Identifying the required minimum sample size for a diagnostic test is necessary to ensure sufficient statistical power to determine diagnostic accuracy within tight confidence intervals [121]. An adequately powered biomarker research study can confidently rule out the possibility that identified markers are chance findings. 

## 5. Conclusions

Several blood- and tissue-based biomarker candidates for EC detection have been reported, however none have yet been translated into routine clinical use. Selection of the right patient groups, consistent sample preparation, and appropriate analytical techniques are crucial for the discovery of clinically relevant biomarkers. While body fluids such as blood are limited by the low amount of cancer-derived proteins in the early phases of EC, tissue specimens are limited by the invasiveness and unacceptability of current sampling techniques. Studies developing valuable biomarkers for EC detection should utilise the potential of high-throughput proteomics on proximal fluids (endometrial fluids) sampled using non-invasive methodologies.

## Figures and Tables

**Table 1:** Potential sources of endometrial cancer (EC) diagnostic biomarkers, their advantages and disadvantages.

Potential Sources of EC Biomarkers	Description	Advantages	Disadvantages
Blood (serum/plasma)	Blood drawn into sample collection tubes.	Easily accessible Minimally invasive High acceptability to both clinicians and patients.	Challenging matrix for proteomic analysis High protein dynamic range Low cancer derived proteins in early phases of disease. Poor concordance with tissue-derived proteins
Hysterectomy specimens	Tissue specimens obtained following hysterectomy	Viable source of biomarkers Relatively low protein dynamic range Good matrix for proteomic analysis	Highly invasive Low acceptability Not feasible for pre-treatment diagnosis
Pipelle biopsy specimens	Endometrial sampling by insertion of the pipelle into the uterine cavity either blindly or at hysteroscopy	Viable source of biomarkers Minimally invasive Relatively low protein dynamic range Relatively good matrix for proteomics Useful in both symptomatic and asymptomatic women.	Severe pain in up to 25% May miss focal pathologies High risk of insertion failure (22% in nulliparous, 8% in parous) Infection, bleeding, uterine perforation
Uterine lavage	Saline is introduced into the uterine cavity and returned by aspiration.	Viable source of biomarkers Relatively low protein dynamic range Relatively good matrix for proteomics Useful in both symptomatic and asymptomatic women.	Relatively invasive Discomfort and pain Low acceptability especially in asymptomatic women
Pap Smear/cervical scrape	A cervical brush is used to sample the ecto-cervix and the endocervical canal.	Simple and minimally invasive Low cost Widely acceptable Viable source of biomarkers	Discomfort from speculum examination Intimate procedure Less useful in asymptomatic women
Tao Brush biopsy specimens	The Tao brush is inserted into the uterine cavity and used to obtain tissue specimens	Less discomfort than pipelle biopsy Viable source of biomarkers Relatively low protein dynamic range	High cost High risk of insertion failure (20% nulliparous, 8% in parous)
Vaginal tampons/swabs	Vaginal tampons used for 8–12 h	Minimally invasive Potential source of uterine biomarkers in symptomatic women	Unappealing to postmenopausal women Inadequate for EC detection in women without bleeding symptoms
Urine	Usually self-collected	Cheap, simple, non-invasive High level of acceptability Can be collected at home/in privacy Relies on renal excretion of systemic biomarkers or urinary contamination by uterine biomarkers Proteins and peptides are stable in urine and less complex	Biomarkers may not be excreted in urine Urinary contamination by uterine biomarkers may be unreliable especially in asymptomatic or minimally symptomatic women Wide variability in urinary protein concentrations

**Table 2:** Summary of important blood-based protein biomarker candidates for EC detection.

Potential Biomarker	Gene Names	Summary of Evidence	Proteomic Techniques Used	Known Biochemical Function	Limitations	Panels
**Serum Amyloid A** **(SAA)**	**SAA1**	**Inconsistent evidence** Upregulated in EC in some studies [60,61]. No difference between cases and controls in others [62,63].	Isobaric tags for relative and absolute quantification (iTRAQ) technology and 2-dimensional liquid chromatography coupled to tandem mass spectrometry (LC–MS/MS). Particle-enhanced immunonephelometry and LS–MS/MS.	High density lipoprotein. Modulates inflammation. Metabolism and transport of cholesterol. Acute phase reactant	Lacks selectivity as also elevated in lung, colon and other cancers	**SAA+HE4** had 73.3% sensitivity and 64% specificity [62]
**Prolactin** **(PRL)**	**PRL**	**Consistent but limited evidence** Sensitivity of 98.3% and specificity of 98% [57]. Upregulated in EC with 16.3% sensitivity and 100.0% specificity at PRL >30 ng/mil [64].	Multiplex xMAP^™^ bead-based immunoassay Electrochemoluminescence immunoassay	Single-chain protein closely related to GH Secretion by stromal cells in response to tumour growth and differentiation Cytokine with immune and inflammatory functions.	Elevated in ovarian, pancreatic and lung cancers.	**Prolactin, GH, TSH, eotaxin and E-selectin** had better accuracy [57]
**Human Epididymis Protein 4** **(HE4)**	**WFDC2**	**Consistent (evidence from meta-analysis)** Sensitivity and specificity of 0.65 (95% CI 0.56–0.73) and 0.9 (95% CI (0.8–0.95) respectively [55].	Enzyme immunoassay, Microparticle immunoassay Electrochemiluminescence	A member of the Whey acidic protein family, located on chromosome 20q 12–13 and acts as a proteinase inhibitor (trypsin inhibitor properties). Possible role in sperm maturation	Expressed in ovarian, renal, lung, colon and breast cancers Suboptimal sensitivity Methodological heterogeneity across studies.	**HE4+CA125** 57–76% sensitivity and 90–100% specificity [65,66,67,68]
**Alpha-1-beta-gylycoprotein** **(AIBG)**	**AIBG**	**Limited evidence** Upregulated in EC [69]	2-Dimensional gel electrophoresis	Plasma glycoprotein encoded by *A1BG* gene with unknown function.	Limited evidence Few studies, obsolete technique	None
**Complement factors** **(C3, C4A, C4B)**	**C3, C4A, C4B**	**Limited evidence** Upregulated in EC [70]	LC–ESI–QTOF(MS1)	Complement proteins involved in immunity and tolerance.	Limited evidence Few studies, small sample size High abundance proteins with low specificity for EC.	None
**Cancer Antigens** **CA125 CA72.4,** **CA15-3**	**MUC 16** **TAG-72** **MUC 1**	**Consistent evidence** CA125 (MUC 16) sensitivity of 17.8–52.6% and specificity of 33.35% to 95% [37].	Enzyme-linked immunosorbent assay (ELISA) Electrochemiluminescence Multiplex bead-based immunoassay	Mucin family glycoprotein, a component of the female reproductive tract, respiratory and ocular surfaces.	Sub-optimal diagnostic accuracy. Elevated in several other malignancies such as ovarian and pancreatic cancers.	See above
**Apolipoproteins (A-IV), C1**	**APOA1-4** **APOC1**	**Limited and inconsistent evidence** Apo A-IV Downregulated in EC [60,70,71]. AI showed SEN 78% and SPE 90%. Apo C1 Upregulated (SEN 82%, SPE 86%) [71].	LC–ESI–QTOF (MS1)/SELDI TOF (MSI) SELDI TOF (MSI)	Lipid transport proteins, stabilise lipoprotein structure and act as enzyme cofactors.	Sub-optimal diagnostic accuracy. High abundance blood proteins with low specificity for EC.	**ApoA-1+TTR+TF** 71% SEN, 88% SPE [72]
**Clusterin** **(CLU)**	**CLU**	**Limited** Upregulated in EC [69]	2 DE Electrophoresis	Also known as Apolipoprotein J, Chaperone with anti-apoptotic properties, involved in preventing the aggregation of non-native protein	Limited evidence. Involved in many conditions related to oxidative stress such as ageing, cancers, neuro- degenerative diseases.	None
**Antithrombin III** **(SERPINC I)**	**SERPINC 1**	**Limited** Upregulated in EC [69]	2 DE Electrophoresis	Glycoprotein produced by the liver, involved in the coagulation system. May inhibit angiogenesis.	Limited evidence, low specificity for EC.	None
**Alpha 1 antitrypsin** **(SERPINA1)**	**SERPINA1**	**Limited** Downregulated in EC [69]	2 DE Electrophoresis	A serine protease inhibitor, inhibits enzymes such as trypsin and neutrophil elastase, produced in the liver and transported to the lungs	Limited evidence, low specificity for EC.	None
**Human chitinase-3 like protein1 (YKL-40)**	**CHI3L1**	**Consistent, limited** Upregulated in EC, 76% sensitivity, 89% specificity [73,74].	Enzyme-linked immunosorbent assay (ELISA)	Glycoprotein of the chitinase family, involved in degradation of extracellular matrix.	Nonspecific. Elevated in colorectal, breast, leukaemia, lung, melanoma cancers, rheumatoid arthritis etc.	None
**Dickkopf-related protein 3 precursor** **(DKK3)**	**DKK3**	**Limited, inconsistent** Upregulated in EC [75] No difference between cases and controls [74]	Enzyme-linked immunosorbent assay (ELISA)	Member of the Wnt signalling pathway important in cell division, formation and cell death during embryogenesis. Reported pro-angiogenic effect in tumour growth.	Limited and inconsistent evidence. Implicated in bone disease, cancer and Alzheimers’ disese.	None
**Visfatin** **(NAMPT)**	**NAMPT**	**Limited** Upregulated in EC, 14.9± 10.6 ng/mL and 8.1± 6,9 ng/mL in EC vs. controls respectively (p:0.011) [76]	Enzyme-linked immunosorbent assay (ELISA)	Secreted by visceral fat, mimics insulin. Possible involvement in metabolic pathways, immune response and cancers.	Limited evidence, may be surrogate for EC risk factors.	None
**VEGFA, VEGFVEGFC**	**PDGFC**	**Limited, Inconsistent** Upregulated in EC [77,78] in some studies, Down regulated in EC [57] in others.	Enzyme-linked immunosorbent assay (ELISA)	Endothelial cell growth factor involved in physiological and pathological angiogenesis.	Limited and inconsistent evidence, non-specific, elevated in many physiological and pathological states.	None
**TSH, ACTH, FSH**	**CGA, POMC, CGA**	**Limited** TSH and ACTH upregulated in EC while FSH is downregulated [57]	Enzyme-linked immunosorbent assay (ELISA), multiplex bead based immunoassay.	TSH and ACTH: communication between immune cells and regulation. FSH: Glycoprotein regulating growth and reproductive processes.	Limited evidence, all non-specific.	**Prolactin, GH, TSH, eotaxin and E-selectin** had better accuracy [57]
**ICAM1/CD54**	**ICAM 1**	**Limited** Upregulated in EC [79]	Flow cytometry	Leucocyte-endothelial transmigration	Limited evidence	None
**Interleukin s/receptors** **(IL31, 33, IL2R)**	**IL31, IL33,** **IL2R**	**Limited** Upregulated in EC [57]	Enzyme-linked immunosorbent assay (ELISA), multiplex bead based immunoassay.	Protein expressed on the surface of immune cells and response to cytokines.	Limited evidence	None
**Cyclophilin A** **(CypA)**	**PPIA**	**Limited** Upregulated in EC [80,81]	Two-dimensional gel electrophoresis and MALDI-Q-TOF MS/MS	Ubiquitous protein, ubiquitous protein, regulates protein folding and trafficking. Plays role in malignant transformation.	Limited evidence, non-specific, high abundance protein, increases with aging and pro-inflammatory conditions such sepsis.	None
**Sperm associated antigen-9**	**SPAG9**	**Limited** Upregulated in EC vs. controls: 18.3 (12.7–53.8) vs. 14.1(4.3–65.3); SEN 70.4% & SPE 82.5% at SPAG9 > 17 ng/mL [82]	Enzyme-linked immunosorbent assay (ELISA).	Scaffold protein involved in signalling pathways, Expressed in testicular haploid germ cells, implicated in infertility.	Limited evidence, non-specific, elevated in cervical, bladder and lung cancers.	None
**Growth-regulated oncogene alpha (CXCL1)**	**CXCL1**	**Limited** Upregulated in EC vs. controls (145(70–270)/90(45–237), *p* < 0.001. AUC = 0.80 [83]	Immunoassay	Chemokine involved in inflammation and tumorigenesis.	Non-specific, elevated in colorectal, melanoma, gastric cancer, ovarian cancer etc.	None
**Growth differentiation factor 15 (GDF-15)**	**GDF15**	**Limited** Upregulated in EC vs. controls. AUC 0.86 [84]	Immunoradiometric sandwich assay with polyclonal goat antihuman GDF-15 antibodies.	A transforming growth factor involved in tissue differentiation and maintenance.	Nonspecific, elevated in ovarian thyroid, pancreatic and colon cancers	None
**Adiponectin, Leptin**	**ADIPOQ** **LEP**	**Consistent** Adiponectin: Downregulated in EC vs. control (mean g/mL 11.3 vs. 17.2 (*p* < 0.0001) [85] Leptin: Upregulated in EC vs. control, mean ng/mL (19.5 vs. 13.4, *p* = 0.03) [85]	Enzyme-linked immunosorbent assay (ELISA) and RIA	Adipokines with metabolic, inflammatory and immune functions. Leptin is pro-inflammatory and adiponectin is anti-inflammatory.	Markers of obesity and metabolic syndrome. Non-specific.	None
**FAS** **(APO1, CD95)**	**FAS**	**Limited** Upregulated in EC [86].	Enzyme-linked immunosorbent assay (ELISA) and RIA	Fas-Fas ligand system important in CTL and NK mediated apoptosis.	Limited evidence	None
**Leucine-rich glycoprotein** **(LRG1)**	**LRG1**	**Limited** Upregulated in EC vs. controls [69].	2 DE Electrophoresis	Involved in protein-protein interaction signal transduction, cell adhesion and neovascularization.	Limited evidence, non-specific.	None
**Matrix metalloproteinase 2,7,9**	**MMP2** **MMP7** **MMP9**	**Inconsistent, limited** MMP7 upregulated in EC, MMP2 and MMP9 downregulated [87].	Enzyme-linked immunosorbent assay (ELISA), multiplex bead based immunoassay.	Enzymes involved in the degradation of extracellular matrix proteins during organogenesis, growth and tissue turnover.	Limited evidence	None
**Transthyretin** **(TTR)/Transferin (TF)**	**TTR** **TF**	**Limited evidence** Upregulated in EC [88]	Immunoassay	TTR: Transport protein that carries thyroid hormone and retinol-binding protein.TF: Iron-binding glycoprotein	Limited evidence, non-specific, associated with amyloidosis, cardiomyopathy etc.	**TTR+TF+** **ApoA:**71%SEN &88% SPE
**Inter-alpha-trypsin inhibitor family heavy chain-related protein (HRP)**	**ITIH4**	**Limited evidence** Upregulated in EC [69]	2 DE Electrophoresis LC-ESI-QTOF (MS1)	Plasma glycoprotein, Serine protease inhibitors.	Limited evidence, non-specific. Dysregulated in multiple solid tumours.	None
**Cleaved high molecular weight kininogen**	**KNG1**	**Limited evidence** Down-regulated in EC vs. Controls [69,89].	2 DE Electrophoresis ITRAQ technology and 2D LC–MS/MS.	Multifunctional plasma proteins involved in the blood coagulation cascade.	Limited evidence, non-specific, high abundance plasma proteins.	None
**Tumor necrosis factor receptor 1A (TNFRSF1A)**	**TNFRSF1A**	**Limited evidence** Upregulated in EC [57].	Enzyme-linked immunosorbent assay (ELISA).	A ubiquitous receptor binding TNF, activating the NF-KB transcription factor, mediating apoptosis and regulating inflammation.	Limited evidence, few studies, not specific elevated in multiple sclerosis, dementia, schizophrenia etc.	None
**Colony stimulating factor 1(CSF1)**	**CSF1**	**Limited evidence** Upregulated in EC vs. Controls [90].	Enzyme-linked immunosorbent assay (ELISA).	Regulatory cytokine involved in the proliferation and differentiation of haematopoietic stem cells.	Limited evidence, few studies, non-specific.	None
**Alpha fetoprotein** **(AFP)**	**AFP**	**Limited evidence** Downregulated in EC [64]	Electrochemiluminescence	Plasma protein whose function in adult humans is less clear. Prevents transport of estradiol across placenta in rodents.	Limited evidence, non-specific, elevated in hepatic cancers germ cell tumours etc.	None

**Table 3:** Summary of important uterine tissue based protein biomarker candidates for EC detection.

Potential Biomarker	Gene Names	Summary of Evidence	Proteomic Techniques Used	Known Biochemical Function	Limitations	Panels
**Chaperonin 10** **(CPN 10)**	**HSPE1**	**Consistent** Upregulated in EC tissues [89,92,94,95,96,103]	iTRAQ and cleavable isotope coded affinity tags (ciCAT) labelled LC-Tandem MS SELDI QTOF MSI	Chaperones involved in normal protein folding, cell signalling and maintenance of the conformation of transduction complexes	Heat-shock proteins are elevated in many other conditions.	**CPN 10, PK and SERPINA1 had SEN, SPE and PPV of 0.95.**
**Calcyphosine** **(CAPS)**	**CAPS**	**Limited** Upregulated in EC tissues [80,81,104].	2 DE Electrophoresis+MS Immunobloting immunohistochemistry	A phosphorylated substrate for cAMP-dependent protein kinase cross-signalling regulating proliferation and differentiation	Limited evidence	None
**Pyruvate Kinase** **(PK)**	**PKM**	**Accumulating evidence** Upregulated in EC tissues [89,93,96,104]	iTRAQ and ciCAT labelled LC-Tandem MS	Regulatory function in the glycolytic pathway	Non-specific, elevated in other malignant and metabolic conditions.	**CPN10, PKM2, SERPINA1 had SEN 0.85, SPE 0.93, PV 0.90.** [93]
**Cyclophilin A** **(CYP A)**	**PPIA**	**Limited evidence** Upregulated in EC by up to 27.23 fold [80,81]	2 DE Electrophoresis+MS Immunobloting immunohistochemistry	Protein folding and immune regulation. Exogenous CYPA may enhance cancer growth via interaction with CD147 and activation of ERK1/2 and MAPK pathways.	Upregulated in lung, pancreatic, hepatocellular and buccal squamous cell carcinomas.	None
**Calgizzarin** **(S-100A11)**	**S-100A11**	**Limited evidence** Upregulated in EC [89,103]	iTRAQ and ciCAT labelled LC-Tandem MS	Calcium binding protein which has roles in cell growth, apoptosis and low grade inflammation.	Non-specific.	None
**Epidermal fatty acid binding protein** **(EFBP)**	**FABP5**	**Limited evidence** E-FABP was upregulated by up to 6.56 fold in EC cases compared to controls [80,81].	2 DE Electrophoresis+MS Immunobloting immunohistochemistry	Fatty-acid binding protein involved in cellular signalling and influences gene expression, growth regulation and cell differentiation.	Up-regulated in oesophageal squamous cell cancer and down-regulated in less differentiated bladder cancer	None
**Calgranulin A** **(S100A8)**	**S100A8**	**Limited evidence** Upregulated in EC [94,103]	MALDI-TOF-MS SELDI-QTOF MSI	S-100 calcium binding protein expressed in multiple cell types. Act as calcium sensors and modulate inflammation.	Limited evidence, non-specific.	None
**Other heat-shock proteins** **HSP27** **HSP47**	**HSPB1** **SERPINH1**	**Limited evidence** Upregulated in EC tissues vs. controls [88,105]. Downregulated in EC [81,104]	2 DE Electrophoresis MALDI Q-TOF MS/MS	Protein folding, cell signalling and maintenance of the conformation of transduction complexes, cell proliferation and differentiation.	Non-specific, limited evidence.	None
**Prohibitin** **(PHB)**	**PHB**	**Limited evidence** Upregulated in EC tissues vs. Controls [88,106].	2 DE Electrophoresis LC–MS/MS	Inhibits DNA synthesis and regulates proliferation.	Limited evidence, few studies.	None
**Transgelin** **(TAGLN)**	**TAGLN**	**Limited evidence** Downregulated in EC [89,96]	iTRAQ and ciCAT labelled LC–Tandem MS	Involved in actin cross linking and protein gelling. Found in many fibroblasts and smooth muscle.	Limited evidence, few studies.	None
**Phosphoglycerate kinase** **(PGK1)**	**PGK1**	**Limited evidence** Upregulated in EC [88,107]	2 DE Electrophoresis + MS Immunohistochemistry	Regulatory enzyme in the glycolytic pathway.	Limited evidence, non-specific	None
**Creatine kinase B**	**CKB**	**Limited evidence** Downregulated in EC [89,96,104]	iTRAQ and ciCAT labelled LC–Tandem MS	Mainly expressed in brain and smooth muscles including vascular and uterine. Major role in energy transduction.	Limited evidence, non-specific	None
**Serotransferrin precursor/Transferrin**	**TF**	**Limited evidence** Upregulated in EC [88] Downregulated in EC [81,104].	2 DE Electrophoresis MALDI Q–TOF–MS/MS	Iron-binding plasma protein.	Limited evidence	None
**Heterogeneous nuclear ribonucleoproteins** **(A2/B1,D0)**	**HNPRNPA1**	**Limited evidence** Upregulated in EC [88,89].	iTRAQ and ciCAT labelled LC-Tandem MS 2 DE Electrophoresis	Protein complexes of RNA important in cell-cycle processes and DNA damage.	Limited evidence	None
**Macrophage migratory inhibitory factor** **(MIF)**	**MIF**	**Limited evidence** Upregulated in EC [89,96].	iTRAQ and ciCAT labelled LC–Tandem MS	Important regulator of the cell-mediated immunity and inflammation.	Limited evidence	None
**Polymeric immunoglobulin receptor precursor** **(PIGR)**	**PIGR**	**Limited evidence** Upregulated in EC vs. Controls [89,96].	iTRAQ and ciCAT labelled LC–Tandem MS	A receptor that binds polymeric IgA and IgM on basolateral surface of epithelial cells. Important in signalling and immunoglobulin transcytosis.	Limited evidence	None
**Alpha-1-antitypsin precursor** **(AIAT)**	**SERPINA1**	**Limited evidence** Down regulated in EC tissues [89,96].	iTRAQ and ciCAT labelled LC–Tandem MS	A serine protease inhibitor, inhibits enzymes such as trypsin	Limited evidence	None
**Capping Actin Protein, Gelsolin Like** **(CAPG)**	**CAPG**	**Limited evidence** Upregulated in EC tissues [104,108]. Downregulated in EC [81]	2 DE Electrophoresis + LC–MS/MS	Actin-based motility in non-muscle cells.	Limited and inconsistent evidence	None
**Protein Deglycase** **(DJ-1)**	**PARK7**	**Limited evidence** Upregulated in EC [108]	2 DE Electrophoresis + LC–MS/MS	Redox-sensitive chaperone and sensor for oxidative stress.	Limited evidence	None
**Annexin-1,2** **(ANXA 1,2)**	**ANXA1, ANXA2**	**Limited evidence** Upregulated in EC [104,106,108]	2 DE Electrophoresis +LC–MS/MS Western blotting Tissue microarray	Bind to cellular membranes in a calcium-dependent manner, mimic glucocorticoid function and exhibits anti-inflammatory properties.	Limited evidence	None
**Peroxiredoxin-1,4** **(PRD-X1,X4)**	**PRDX1-4**	**Limited evidence** Upregulated in EC [81,106]	LC–MS/MS Western blotting Tissue microarray	Scavenging of peroxides, protection from oxidative stress-induced apoptosis.	Limited evidence	None
**Costars family protein ABRACL**	**ABRACL**	**Limited evidence** Upregulated in EC [101]	2 DE Electrophoresis LC–MS/MS Western blotting	ABRACL is an 82 amino acid protein that regulates actin cytoskeleton dynamics and motility.	Limited evidence	None
**Phosphoglycerate mutase 2** **(PGAM2)**	**PGAM2**	**Limited evidence** Upregulated in EC [101]	2 DE Electrophoresis LC–MS/MS Western blotting	Glycolytic enzyme modulating NADPH homeostasis, impacting cell proliferation and tumour growth.	Limited evidence	None
**Glutathione synthetase** **(GSS)**	**GSS**	**Limited evidence** Upregulated in EC [101]	2 DE Electrophoresis LC–MS/MS Western blotting	Cellular homeostasis and anti-oxidant properties.	Limited evidence	None
**Desmin** **(Des)**	**DES**	**Limited evidence** Downregulated in EC [81,105].	DIGE MALDI–TOF	Protein marker for muscle tissue	Limited evidence	None
**Alpha enolase** **(ENO1)**	**ENO1**	**Limited evidence** Upregulated in EC 220 fold [100,101,106]	2 DE Electrophoresis LC–MS/MS Western blotting	Glycolytic enzyme. Regulates the PI3K/AKT signalling pathway and induces tumorigenesis by activating plasminogen.	Limited evidence	None
**Superoxide dismutase** **(SOD1&2)**	**SOD1** **SOD2**	**Limited evidence** SOD2 Upregulated in EC 5 fold [101]. SOD1 downregulated in EC [105]	2 DE Electrophoresis LC–MS/MS Western blotting	Has anti-apoptotic effects against oxidative stress, ionizing radiation, and inflammatory cytokines.	Limited evidence	None
**Fibrinogen beta chain** **(FBG)**	**FBG**	**Limited evidence** Upregulated in EC up to 400 fold [101].	2 DE Electrophoresis LC–MS/MS Western blotting	Blood-based glycoprotein	Limited evidence	None
**Anterior Gradient 2 protein**	**AGR2**	**Limited evidence** Upregulated in EC [81,104]	2 DE Electrophoresis MALDI Q–TOF MS/MS	A protein disulphide isomerase involved in protein folding and implicated in various cancers.	Limited evidence	None
**Clusterin** **(CLU)**	**CLU**	**Limited evidence** Upregulated in EC [96]	LC + Tandem MS/MS	Chaperone with anti-apoptotic properties.	Limited evidence	None
